# Comparison of intraoperative frozen section consultation and hysterectomy characteristics in patients diagnosed with EIN in endometrial biopsies

**DOI:** 10.3389/pore.2025.1612039

**Published:** 2025-06-05

**Authors:** Hasan Volkan Ege, Bilal Esat Temiz, Alp Usubutun, Deniz Ates Ozdemir, Muhammed Onur Atakul, Murat Cengiz, Utku Akgor, Derman Basaran, Murat Gultekin, Mehmet Coskun Salman, Zafer Selcuk Tuncer, Nejat Ozgul

**Affiliations:** ^1^ Division of Gynecologic Oncology, Department of Obstetrics and Gynecology, Faculty of Medicine, Hacettepe University, Ankara, Türkiye; ^2^ Gynecology and Obstetrics Clinic, 29 Mayıs State Hospital, Ankara, Türkiye; ^3^ Department of Pathology, Faculty of Medicine, Hacettepe University, Ankara, Türkiye; ^4^ Department of Obstetrics and Gynecology, Faculty of Medicine, Hacettepe University, Ankara, Türkiye; ^5^ Division of Gynecologic Oncology, Department of Obstetrics and Gynecology, Baskent University, Ankara, Türkiye

**Keywords:** endometrial cancer, FIGO 2023, frozen-section assessment, endometrioid intraepithelial neoplasia, endometrial hyperplasia

## Abstract

**Objective:**

This study aimed to assess the likelihood of detecting cancer in final pathology and evaluate the accuracy of intraoperative frozen-section assessment in cases of endometrioid intraepithelial neoplasia (EIN).

**Material and methods:**

We included patients diagnosed with EIN at Hacettepe University Hospital who subsequently underwent hysterectomy at the same center between January 2011 and March 2023. EIN diagnoses made at other institutions were re-evaluated and confirmed by co-author gynecopathologists.

**Results:**

A total of 354 patients diagnosed with EIN underwent hysterectomy. The majority of patients (68.5%) had a final diagnosis of EIN. Endometrial cancer (EC) was identified in 11.3% (n = 40) of patients in the final pathology. Advanced age (≥50 years) (OR = 2.52; 95% CI: [1.27–4.96]; p = 0.006) and menopausal status (OR = 2.62; 95% CI: [1.34–5.11]; p = 0.004) were significantly associated with an increased risk of EC. Among 263 patients who underwent intraoperative frozen-section assessment, EC was detected in 12.9% (n = 34). The sensitivity and specificity of frozen-section assessment for EC detection were 41.1% and 100%, respectively. The frozen-section assessment failed to identify only one of the seven patients who required staging surgery.

**Conclusion:**

Our study demonstrates that a preoperative EIN diagnosis carries an 11.3% risk of concurrent EC. Additionally, the likelihood of EC is significantly higher in older and postmenopausal patients. The majority of patients requiring staging surgery were identified by frozen-section assessment. Our findings indicate that frozen-section assessment provides the necessary information for adequate surgical treatment in EIN cases.

## Introduction

Endometrial cancer (EC) is the second most common malignancy of the female genital tract and the most frequently diagnosed gynecologic cancer in developed countries [1]. The predominant histologic subtype, endometrioid adenocarcinoma, arises from well-defined precancerous lesions, most notably atypical endometrial hyperplasia, also referred to as endometrioid intraepithelial neoplasia (EIN) [2, 3]. Identifying these precursor lesions provides an opportunity for early intervention, potentially reducing EC-related morbidity and mortality.

EIN is clinically significant due to its risk of concurrent EC at diagnosis and its potential for progression to invasive cancer [4–6]. The reported rate of concomitant EC in EIN cases varies between 30% and 50% across studies [7–12]. In a meta-analysis of 15 trials, the overall pooled rate of concurrent EC in endometrial hyperplasia was 32.1% (range 5.9%–53.1%) [8]. While most EC cases associated with EIN are low-stage and low-grade, some patients present with high-risk disease, necessitating lymph node dissection (LND) for proper staging and treatment.

The decision to perform staging surgery, including LND, is often guided by intraoperative frozen-section assessment. However, its accuracy varies, and studies report inconsistent agreement between frozen-section results and final pathology, potentially leading to misclassification of patients requiring LND [6, 10, 13].

This study aims to determine the likelihood of concurrent EC in EIN lesions and identify clinical factors associated with an increased cancer risk. Additionally, we evaluate the effectiveness of intraoperative frozen-section assessment and explore factors influencing its diagnostic accuracy.

## Materials and methods

This retrospective study included patients diagnosed with EIN who subsequently underwent hysterectomy at Hacettepe University Hospital between January 2011 and March 2023. Demographic, clinical, and histopathological data, including all pathology reports, were retrieved retrospectively through electronic medical records and archival review.

All EIN diagnoses were confirmed by gynecopathologists (AU, DAO) affiliated with our institution. The study population comprised patients diagnosed with EIN either from endometrial samples obtained at our center or from paraffin-embedded tissue preparations referred from external institutions. For externally obtained preparations, histologic slide reviews were performed, and diagnoses were verified by the co-author gynecopathologists. The diagnosis of EIN was established based on subjective histopathological criteria [14]. Our institution has consistently applied the current EIN diagnostic criteria since 2014.

All patients included in the study underwent total hysterectomy. The decision to perform intraoperative frozen-section assessment of hysterectomy specimens was at the discretion of the operating surgeon. In cases where malignancy was identified intraoperatively, staging surgery was performed based on the surgeon’s clinical judgment. The decision to perform bilateral salpingo-oophorectomy was individualized, considering factors such as patient age, clinical indications, and patient preference.

Final pathology evaluations were conducted by the same team of gynecologic pathologists. As a standard institutional practice, if no gross tumor was identified in the hysterectomy specimen, the entire endometrium was sampled to minimize the risk of missing an underlying carcinoma. The presence of tumors smaller than 1 mm was classified as microscopic disease. EC cases were staged according to both the 2009 and 2023 International Federation of Gynecology and Obstetrics (FIGO) staging systems.

A comparative analysis was performed to assess concordance between preoperative endometrial biopsy results, intraoperative frozen-section findings, and final pathology outcomes.

The study was approved by the Health Sciences Research Board of Hacettepe University (Decision Number: 2023/02-28, Approval Date: September 12, 2023).

Statistical analyses were conducted using SPSS version 23 (IBM Corp., Armonk, NY, USA). Numerical variables were reported as medians, based on their distribution assessed by the Kolmogorov-Smirnov and Shapiro-Wilk tests. Comparisons between numerical variables were performed using the Mann-Whitney U test or the independent-samples t-test, as appropriate. Categorical variables were presented as frequencies and percentages, with comparisons conducted using the Chi-square test. A p-value of <0.05 was considered statistically significant.

## Results

A total of 413 patients were diagnosed with EIN at our center. Of these, 354 patients underwent at least a hysterectomy. The median age at the time of surgery was 49 years (range: 31–86), and the median body mass index (BMI) was 29.9 kg/m^2^ (range: 17.5–57.1). The most prevalent comorbidity was diabetes mellitus (DM), which was present in 28% of cases. At the time of surgery, 31.9% of patients (n = 113) were postmenopausal. The majority of patients (78.8%) underwent surgical intervention within 4 weeks of diagnosis. Bilateral salpingo-oophorectomy was performed in 92.6% of patients who underwent hysterectomy.

**FIGURE 1:**
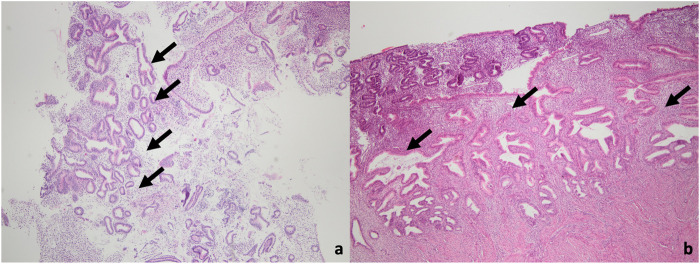
Microscopic images of the case diagnosed with EIN in the final pathology. **(a)** A small focus of EIN (arrow) in the curettage material (H&E, ×40 magnification). **(b)** A focal EIN focus (arrow) also in hysterectomy material (H&E, 40x magnifcation).

EC was identified in 11.3% of patients (n = 40) in the final pathology reports. The majority of patients (68.5%) had a final diagnosis of EIN (Figure 1). Based on the FIGO 2009 staging system, most patients with EC (87.5%, n = 35) were classified as stage IA (Table 1). According to the FIGO 2023 staging system, the majority were categorized as stage IA1 (Table 1). Among patients diagnosed with EC, 23 cases had disease confined to the endometrium. Myometrial invasion of 50% or greater was observed in four patients.

**TABLE 1:** Demographic and clinicopathological characteristics of patients with EIN (n = 354).

Parameters	All patient (n:354)	Endometrial cancer positive (n:40)	Endometrial cancer negative (n:314)	p-value
Age (years) * <50 ≥50	49.3 ± 8.0 204 (57.6%) 150 (42.4%)	52.6 ± 8.7 15 (37.5%) 25 (62.5%)	48.9 ± 7.8 189 (60.2%) 125 (39.8%)	**0.006** **0.006**
Body Mass Index * <30 ≥30	30.2 ± 5.8 148 (51.2%) 141 (48.8%)	31.45 ± 5.95 12 (41.4%) 17 (58.6%)	30.22 ± 6.02 136 (52.3%) 124 (47.7%)	0.253 0.328
Endometrial Thickness (mm) * <20 ≥20	13.1 ± 5.4 246 (88.5%) 32 (11.5%)	13.56 ± 6.96 26 (81.2%) 6 (18.8%)	13.03 ± 5.22 220 (89.4%) 26 (10.6%)	0.612 0.232
Time between diagnosis and operation (weeks) **	2.0	2.66	3.30	0.686
CA 125* <35 ≥35	25.1 ± 42.0 170 (86.7%) 26 (13.3%)	16.02 ± 12.73 20 (90.9%) 2 (9.1%)	26.34 ± 44.29 150 (86.2%) 24 (13.8%)	0.208 0.744
Menopause Yes No	112 (31.8%) 240 (68.2%)	21 (52.5%) 19 (47.5%)	93 (29.6%) 221 (70.4%)	**0.004**
Parity Yes No	267 (94.1%) 17 (5.9%)	33 (91.7%) 3 (8.3%)	234 (94.4%) 14 (5.6%)	0.461
Diabetes Mellitus Yes No	99 (28.0%) 255 (72.0%)	13 (32.5%) 27 (67.5%)	86 (27.4%) 228 (72.6%)	0.294
Hypertension Yes No	50 (14.1%) 304 (85.9%)	6 (15%) 34 (85%)	46 (14.5%) 270 (85.5%)	0.427
Operations TAH/TLH/VH TAH/TLH/VH + BSO TAH/TLH/VH + BSO + LND	26 (7.3%) 322 (90.9%) 6 (1.8%)	2 (5%) 34 (85%) 4 (10%)	24 (07.6%) 288 (91.7%) 2 (0.7%)	
Stage (FIGO 2009) 1A 1B 2		35 (87.5%) 4 (10%) 1 (2.5%)	N/A	
Stage (FIGO 2023) 1A1 1A2 1B 2A		23 (57.5%) 12 (30%) 4 (10%) 1 (2.5%)	N/A	
Grade 1 2		37 (92.5%) 3 (7.5%)	N/A	
Tumor Size <2 cm ≥2 cm		32 (80%) 8 (20%)	N/A	
Myometrial invasion Limited to the endometrium <½ ≥½		23 (57.5%) 13 (32.5%) 4 (10%)	N/A	
LVSI Yes No		1 (2.5%) 39 (97.5%)	N/A	

Statistically significant results are shown in bold. Abbreviations: TAH, total abdominal hysterectomy; BSO, bilateral salpingo-oophorectomy; LND, lymph node dissection; TLH, Total laparoscopic hysterectomy; VH, Vaginal hysterectomy.

* Mean ± sd, **median.

**FIGURE 2:**
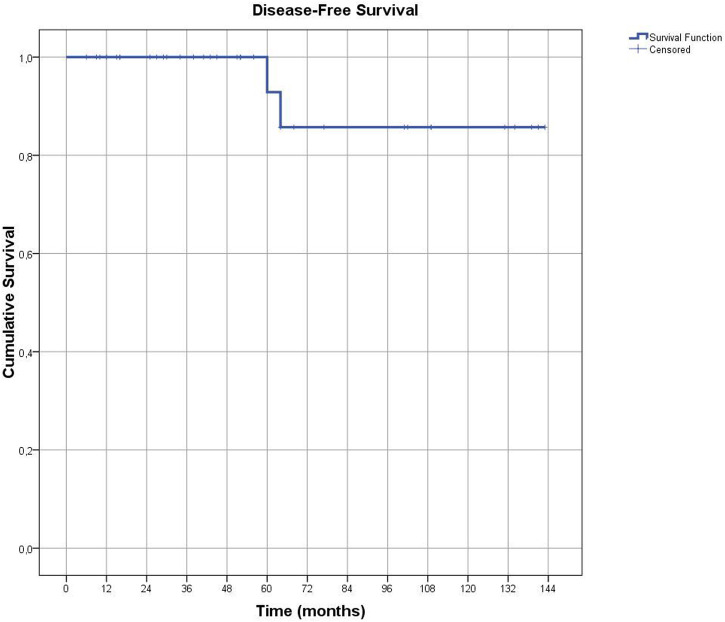
The graph shows Disease-Free Survival in patients diagnosed with endometrial cancer.

Notably, the majority of EC cases were grade 1 (92.5%), with no cases of grade 3 disease identified. Tumor size was less than 2 cm in most patients (80%). Lymphovascular space involvement (LVSI) was assessed in all patients with EC, and focal LVSI was identified in only one patient. Based on the Mayo criteria, lymph node dissection was indicated in nine patients [15]. Omentectomy was performed in 31 patients, including 11 with a final diagnosis of EC, but no cases of tumor metastasis were detected in the omental specimens. The median follow-up duration for the 36 EC patients with available follow-up data was 51.5 months (range: 6–143 months). Patients diagnosed with EC were monitored during routine follow-up visits, which included physical examinations and annual abdominal imaging (ultrasound or computed tomography). Recurrence was identified in only one patient, detected radiologically at 64-months post-surgery. This patient had previously received adjuvant radiotherapy following surgical treatment and was also the only case in the study with focal LVSI (+). Kaplan-Meier analysis demonstrated a median disease-free survival of 131.4 ± 7.5 months among the 40 patients diagnosed with EC (Figure 2).

The groups with and without EC were subjected to a comparative analysis based on age, menopausal status, parity, BMI, obesity, endometrial thickness - before endometrial sampling- (ET), CA 125 levels, and comorbidities (Table 1). Notably, EC prevalence was significantly higher in the postmenopausal group (OR = 2.62; 95% CI: [1.34–5.11]; p = 0.004). Also, the mean age was notably higher in the EC-positive group (p = 0.006). In the group aged 50 years and older, EC was significantly higher in the final pathology (OR = 2.52; 95% CI: [1.27–4.96]; p = 0.006). However, no significant differences were observed between the two groups in other parameters.

Among the 354 patients who underwent surgery, intraoperative frozen-section assessment was performed in 263 cases (74.2%). Within this cohort, EC was identified in 34 patients (12.9%) in the final pathology following frozen-section evaluation. The frozen-section diagnoses demonstrated a 92.4% concordance with final pathology regarding the presence or absence of coexistent carcinoma.

**FIGURE 3:**
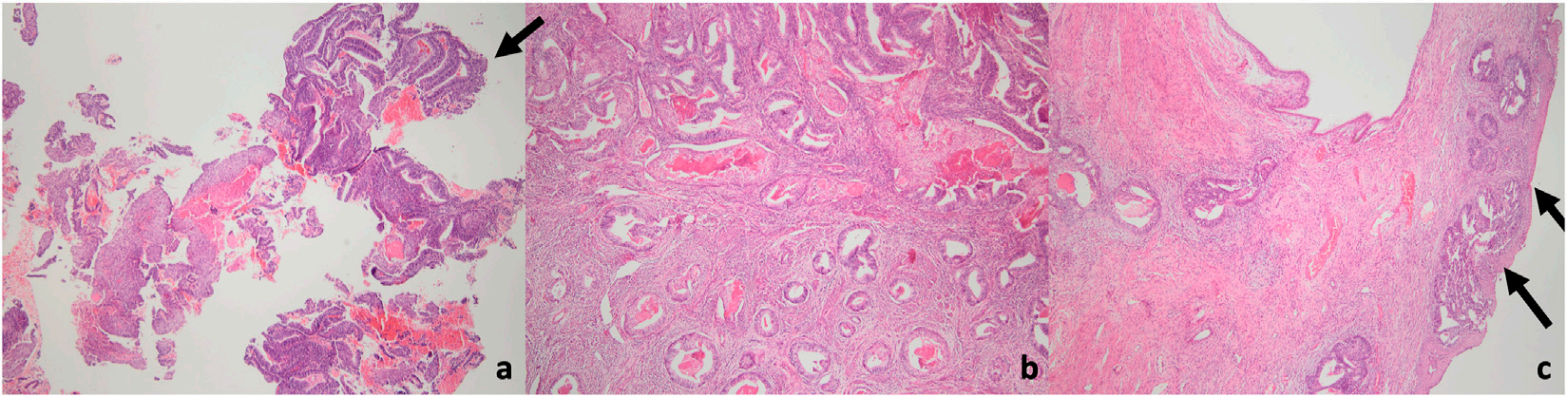
Microscopic images of the endometrial cancer case with cervical stromal invasion detected in the final pathology. **(a)** EIN focus, suspicious for adenocarcinoma (arrow) in a superficial endometrial fragment (H&E, 40x magniifcation). **(b)** Deep invasion extending deep into the myometrium in hysterectomy material (H&E, 40x magniifcation). **(c)** Tumoral invasion under the ectocervix in hysterectomy material (arrow). Endocervical stromal invasion + (H&E, 40x magniifcation).

In 249 cases, frozen-section assessment did not indicate EC. However, in 20 of these cases, a final diagnosis of EC was established upon permanent section analysis. Among these 20 undetected cases, only one had grade 2 disease. In all cases where EC was not identified intraoperatively, tumor size was below 2 cm, with nine patients exhibiting microscopic disease. The disease was confined to the endometrium in 12 cases, while superficial myometrial invasion was observed in eight patients. No cases demonstrated a myometrial invasion depth of 50% or greater. Staging surgery was required in only one case due to cervical stromal invasion (Figure 3).

All 14 patients in whom EC was identified through intraoperative frozen-section assessment were confirmed to have EC in the final pathology results (Table 2). Regarding the presence or absence of concomitant EC, frozen-section diagnoses were concordant with final pathology in 243 out of 263 cases (92.4%).

**TABLE 2:** Intraoperative frozen section results.

Frozen section diagnosis (n)	Final pathology (n)		Total
	Benign/EIN	Malign	
Benign/EIN	229	20	249
Malign	0	14	14
Total	229	34	263

The diagnostic performance of frozen-section assessment for detecting concomitant EC in patients undergoing surgery for EIN demonstrated a sensitivity of 41.1%, specificity of 100.0%, positive predictive value (PPV) of 100.0%, and negative predictive value (NPV) of 91.9% (Table 2).

## Discussion

This study, to our knowledge, is one of the largest single-centre series in Turkey that examines the relationship between EIN and EC to date, including the results of frozen-section assessment in EIN management. Few studies globally have analyzed comparable patient numbers. Our analysis revealed an EC prevalence of 11.3%, significantly lower than the 30%–50% reported in most studies [7, 9–12, 16, 17]. This discrepancy may be attributable to the younger mean age or pre-dominantly premenopausal status of our patient cohort because our gynecology and pathology department gain a lot of consultations. However, some studies in the literature report a concurrent EC rate of less than 10% in cases of endometrial hyperplasia [8].

Our center utilizes the 2000 EIN classification for diagnosing EIN, a system shown to be reproducible despite its subjective criteria [18, 19]. The lower rate of concomitant cancer we observe is likely a result of early phase of the diseases diagnosed by our expert gynecopathology review, which prioritizes accurate identification of precursor lesions and removal of invasive disease from the EIN category. The demographic characteristics of our patient cohort contributed to the low EC rate. Given that before the use of EIN criteria for diagnosis approximately 30% of hyperplasia without atypia (complex or simple) would be diagnosed as EIN [14], and that these lesions are often focal and mild (representing early neoplastic development), this change may inadvertently decrease the detection of concurrent cancers in EIN patients. More studies using the WHO 2020 criteria are crucial to assess this potential impact.

Several studies have reported higher rates of concurrent EC in patients with EIN compared to our cohort. Vetter et al. identified EC in 47% of 169 EIN patients, a disparity likely attributable to demographic differences [10]. Their cohort had a higher median age (56 vs. 49 years), a greater proportion of postmenopausal patients (63.4% vs. 31.9%), a higher prevalence of obesity (82.8% vs. 31.9%), and a greater median BMI (39.5 vs. 29.9) compared to our study. Similarly, Abt et al. reported a 27% EC rate in a predominantly white (74%) patient population with a median age of 56 years, where comorbidities such as hypertension were also more prevalent [11]. Additionally, a recent study observed a 47% EC rate among 98 EIN patients, with a higher mean age (64 vs. 49 years) and a smaller proportion of premenopausal patients (15% vs. 68.2%) than in our study [12]. These demographic differences likely contribute to the observed discrepancies in the rates of concurrent EC.

Our analysis of patients with EC on final pathology (n = 40) compared to those without (n = 314) demonstrated significant differences in mean age (52.6 vs. 48.9 years, p = 0.006) and menopausal status (52.5% vs. 29.6%, p = 0.008). A significantly greater proportion of patients aged 50 and older had concurrent EC (62.5% vs. 39.8%, p = 0.006). These findings indicate that EIN patients aged 50 and older (OR = 2.52, 95% CI: 1.27–4.96) or postmenopausal women with EIN (OR = 2.62, 95% CI: 1.34–5.11) are at increased risk of concurrent cancer. Therefore, a more comprehensive evaluation is warranted in these subgroups, including specialist review of preoperative pathology.

The association between older age and concurrent EC in EIN patients is well-supported by the literature. Abt et al. [11] reported a significantly higher mean age in the EC group (59 vs. 54 years, p = 0.003), a finding corroborated by Havez et.al. [12] (70 vs. 61 years, p < 0.001). Our prior study [6], as well as the current one, found a similar trend, with higher mean age and a greater proportion of postmenopausal patients in the EC group, consistent with other published data [10, 20]. Interestingly, we did not find significant differences in endometrial thickness, hypertension, or parity between the groups, despite their reported association with EC risk in some studies [10, 11, 20].

Endometrioid-type adenocarcinomas are widely accepted to arise from EIN or atypical hyperplasia, typically presenting as well-differentiated disease. Consistent with this, all EC cases in our study were endometrioid, with the majority (92.5%) being grade 1 [5, 6, 12, 17]. The predominance of stage-1 EC in our study, as classified by both FIGO 2009 and 2023, is consistent with established patterns. Our finding of 87.5% of cases in stage-1A closely mirrors the 86.6% reported by Vettel et al. [10]. Abt et al., however, reported a much lower rate of EC (27%) with only 5% beyond stage 1A [11]. This discrepancy may be related to their specific patient population. Havez et al. reported approximately 35% stage >1A disease, which they attribute to a higher rate of synchronous EC (47%), an older median patient age (71), and the use of sentinel lymph node sampling, which can identify metastatic disease and thus increase the apparent proportion of advanced-stage cancers [12].

LVSI has gained increasing significance following the introduction of the FIGO 2023 staging system. In our study, LVSI status was available for all patients diagnosed with EC, with only one patient (2.5%) exhibiting focal LVSI positivity. In comparison, Abt et al. reported an LVSI rate of 3%, while Vetter et al. and Havez et al. observed higher rates of 11% [10–12]. The discrepancies in LVSI rates between studies may be explained by variations in patient demographics and the limited number of EC cases in some cohorts.

Our study also examined the role of frozen-section assessment in EIN management. Although frozen-section was 100% accurate in identifying EC when present (n = 14), it failed to detect a substantial proportion of malignant cases (58.8%). These undetected cases were primarily grade 1 (95%), confined to the endometrium (60%) or with <1/2 invasion (40%), and all were <2 cm. This highlights the limitations of frozen-section in detecting early-stage EC. Despite this, only one of the 20 patients missed by frozen-section required staging surgery. Frozen section, using Mayo criteria, effectively identified 6 of the 7 patients requiring staging, enabling appropriate surgical planning. Our study demonstrates that while frozen-section assessment for EC in EIN has high specificity (100%), PPV (100%), and NPV (91.9%), its sensitivity is limited (41.1%). A study of 128 atypical endometrial hyperplasia cases reported a substantially higher frozen-section EC detection rate (80.3%) [21]. However, their much higher EC prevalence (53.1%) in the final pathology likely accounts for this difference. This highlights the crucial role of EC prevalence when interpreting the sensitivity of frozen-section assessments.

Our findings highlight the challenge of detecting small, millimeter-scale tumors during frozen-section assessment. While this limitation exists, the clinical impact appears minimal, as only one patient in this group required staging surgery. Given the difficulty of distinguishing EIN from invasive EC intraoperatively, careful slide review before surgery is essential. Our staging analysis (FIGO 2009 and 2023, incorporating LVSI data) demonstrated predominantly early-stage EC, with adjuvant treatment needed in only a small fraction of cases. This suggests that surgery alone is often sufficient. Critically, despite the inherent limitations of frozen section, the need for staging surgery was only missed in one patient, underscoring its overall clinical utility.

### Strengths and limitations

While our single-center study highlights the positive influence of gynecopathologists and frozen-section assessment in managing endometrial precancerous lesions (EIN), and presents cancer staging according to the FIGO 2023 system, it is important to acknowledge several limitations. The retrospective design inherently limits the conclusions that can be drawn. Crucially, our data lacks key demographic information potentially associated with EC, including ethnicity, history of cancer, and estrogen use. The absence of a control group limits our ability to definitively quantify the impact of gynecopathologist expertise on EIN diagnosis, a key finding of our study. Similarly, the studies we used for comparison lacked information on the specific expertise of the pathologists involved. Finally, data on POLE mutation status, relevant to the FIGO 2023 staging system in EC patients, is also unavailable.

## Conclusion

This study demonstrates that advanced age (50 years or older) and postmenopausal status are significantly associated with concurrent EC in patients with EIN. Specialized gynecopathology expertise may improve the accurate evaluation and management of EIN, especially in patients desiring future fertility, where hysterectomy should only be considered when all other options are exhausted. Frozen-section assessment provides sufficient information for the surgical management of EIN cases.

## Data Availability

The raw data supporting the conclusions of this article will be made available by the authors, without undue reservation.
